# Sex differences in neuropathological response to traumatic brain injury: increased neuronal loss and astrogliosis in females

**DOI:** 10.1007/s00429-025-02986-6

**Published:** 2025-07-25

**Authors:** Zuzanna Rauk, Joanna Jędrusik, Zofia Walczak, Zuzanna Setkowicz

**Affiliations:** 1 Doctoral School of Exact and Natural Sciences, Jagiellonian University, Prof. St. Łojasiewicza St 11, PL30348 Cracow, Poland; 2 Faculty of Biology, Institute of Zoology and Biomedical Research, Laboratory of Experimental Neuropathology, Jagiellonian University, Gronostajowa 9 St, PL30387 Cracow, Poland

**Keywords:** Traumatic brain injury, Sex differences, Glial scar, Astrocytes, Microglia, Neurons, Cell morphology

## Abstract

Traumatic brain injury (TBI) is one of the most common causes of disability worldwide and a risk factor for the development of post-traumatic epilepsy and mood disorders. Sexual differences in the tissue response to the injury may contribute to the varied pathophysiology of TBI, making it particularly challenging to develop a satisfactory therapy. The aim of this study was to investigate the sexual difference in astrogliosis, microgliosis, and neuronal loss after TBI. Penetrating cortical brain injury was performed in male and female rats that were sacrificed 2, 8, 16, or 30 days after injury. Glial scar development and neuronal loss were analysed, as well as the morphology of astrocytes and microglia in perilesional cerebral cortex. Increased astrogliosis was observed in females compared to males, including more complex and hypertrophied morphology of astrocytes 2 and 8 days after TBI, an earlier onset of contralateral astrocytic reaction, and a greater GFAP + (glial fibrillary acidic protein) area fraction in perilesional cortex in females 30 days post-injury. Sex differences in microglia morphology were also observed, such as more complex and ramified microglia in females 2 and 30 days after TBI. Moreover, an increased loss of parvalbumin- and neuropeptide Y-expressing neurons in perilesional and contralateral cortex was noticed in females compared to males, along with a higher number of cells expressing neuronal nitric oxide synthase. These results suggest a sexual differences in the cellular response to traumatic brain injury, which may contribute to the different outcomes and development of post-traumatic pathologies in males and females.

## Introduction

Traumatic brain injury (TBI), often designated as a ‘silent epidemic,’ is one of the main causes of disability and mortality worldwide (Dewan et al. 2019). This dysfunction occurs as a result of the impact of external forces on the brain, typically related to traffic accidents, falls, and injuries sustained in military and sports activities (Har-Even et al. 2021). The patients frequently suffer from cognitive, behavioural, and emotional impairments and eventually may develop serious neurological disorders such as neurodegenerative diseases or post-traumatic epilepsy. These gradually evolving TBI sequelae result from a multidimensional cellular and molecular response of insulted tissue, including neuronal death, inflammation, or reactive gliosis.

Reactive astrocytes are responsible for restoring homeostasis during the acute stage of TBI, including clearing extracellular glutamate, repairing the blood–brain barrier (BBB), and limiting the inflammatory response (Kawano et al. 2012). However, the microglia-astrocytes crosstalk and their prolonged activation lead to the loss of astrocytic homeostatic function and a chronic inflammatory state, inducing secondary injury and further nervous tissue degeneration. Moreover, the glial scar created by reactive astrocytes constitutes a molecular and mechanical barrier for axonal regrowth and promotes neuronal hyperexcitability, which may lead to the development of an epileptogenic focus (Xu et al. 2019). The complex response of injured tissue makes it particularly challenging to develop a satisfactory therapy. Thus, there is a need for a better understanding of the underlying mechanisms of TBI.

The increasing incidence of TBI in women raises questions about sex differences in TBI pathophysiology. Women patients report more headaches, dizziness, and drowsiness after injury and are more likely to suffer from anxiety and depression as post-injury symptoms than males. Animal studies report better outcomes in females than males, in contrast to human studies that reveal worse outcomes in women than men (Gupte et al. 2019). This discrepancy may result from different sample sizes, injury severities, and experimental designs. Additionally, few studies to date have examined the influence of sex on gliosis in TBI. Therefore, further research is needed to elucidate the reasons for sex differences in TBI and develop therapeutic solutions appropriate for each sex.

In our study we used the rat model of penetrating brain injury, an open, focal TBI with a relatively small degree of tissue damage, but with blood–brain barrier disruption, glial scarring, and epileptogenic potential, which was successfully used in our previous research (Janeczko 1989; Setkowicz and Janeczko 2003). In order to observe the gradual development of glial scar within 30 days post-TBI, certain cohorts of animals were sacrificed 2, 8, 16, and 30 days post-TBI, and histological analysis of reactive gliosis was performed. Moreover, we have investigated the neuronal density in the perilesional cerebral cortex, including parvalbumin- and neuropeptide Y-expressing neurons, crucial for the maintenance of excitation-inhibition balance in neural circuits, as well as the neuronal NO synthase (nNOS)-expressing neurons, as a source of the nitric oxide (NO), a potentially neurotoxic gasotransmitter. This is the first study to investigate the dynamics of reactive gliosis and neuronal loss after TBI in both sexes. The abovementioned analyses allowed us to obtain a complete picture of posttraumatic changes and identify existing sex differences.

## Methods

### Animals

All procedures using experimental animals were approved by the Second Local Ethical Committee in Cracow (agreement no. 316/2020) and were in accordance with the international standards. Seventy four male and 69 female Wistar rats were obtained from the animal colony in the Laboratory of Experimental Neuropathology (Institute of Zoology and Biomedical Research, Jagiellonian University, Cracow, Poland). During this experiment, the rats were bred under the conditions of controlled temperature (20 ± 2 °C) and illumination (12-h light:12-h dark cycle). A solid diet (Morawski Labofeed H Standard) and water were available for them ad libitum.

### Penetrating brain injury

Thirty-day old male and female rats were anaesthetised with inhaled isoflurane mixed with oxygen (Isoflurin, 2–4%). The injury was made according to the procedure described in our previous papers (Kawon et al. 2021; Setkowicz et al. 2016). Briefly, a rotating dental drill (1.2 mm diameter), was inserted in the left brain hemisphere, perpendicularly to the skull surface, to penetrate the whole depth of the cerebral cortex up to the underlying white matter (depth: 2 mm adjusted using a limiting plastic ring fixed on the drill). The location was specified with a stereotactic apparatus; approximate coordinates of the drill tip were as follows: antero-posterior: −0.30; medio-lateral: 3.0; dorso-ventral: 7.2 (Paxinos and Watson 2007). After the procedure, the skin on the rat head was sutured, and the animal was placed back into the cage. The control groups were housed without the TBI induction.

### Tissue fixation and staining procedure

**Fig. 1:**
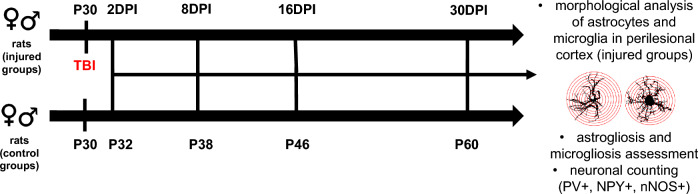
Experimental scheme. Female and male rats underwent penetrating traumatic brain injury on postnatal day 30 (P30) and were sacrificed at 2DPI (days post injury), 8DPI, 16DPI, and 30DPI. Control groups were sacrificed on P32, P38, P46, and P60. Subsequently, brain sections were collected and morphology of astrocytes and microglia, astrogliosis and microgliosis as well as neuronal density were assessed

To observe a gradual development of glial scar formation, certain cohorts of animals were sacrificed at 2, 8, 16, and 30 days post-injury (DPI). Control rats were sacrificed on P32 (postnatal day 32), P38, P46, and P60. The rats received a lethal dose of pentobarbital (Morbital, Biowet) and were perfused transcardially with 0.9% NaCl followed by 4% formalin. After removing from the skull, the medulla was dissected, and the brain was kept at 4 ℃ in 4% formalin. Figure 1 presents the experimental scheme. Animal numbers in the examined control and experimental groups are presented in Table 1.

**Table 1:** Numbers of animals used in the present study

	Females				Males			
	2 DPI	8 DPI	16 DPI	30 DPI	2 DPI	8 DPI	16 DPI	30 DPI
Injured group	8	9	9	9	11	9	9	9
Control group	7	9	9	9	9	10	8	9
Total: 143 rats								

*DPI* days post injury. 2, 8, 16, 30—days post-injury, when animals were sacrificed

For immunostaining, the brains were immersed in TBS and cut with a vibratome (Leica VT1000S) to obtain 30 µm frontal sections. From the injured brains, the sections containing the lesion site, i.e. from the level of anterior commissure, were collected. The sections taken from the control, uninjured brains, represented the same locations. For astroglial and microglial activation and neuronal loss assessment, immunohistochemical stainings for GFAP (glial fibrillary acidic protein; DAKO, Z0334, 1:2000); Iba1 (ionized calcium-binding adapter molecule 1; FujiFilm, 019–19741, 1:2000): PV (parvalbumin, Merck Life Science, MAB1572, 1:2000); NPY (neuropeptide Y, Merck Life Science, N9528, 1:10 000) and nNOS (neuronal nitric oxide synthase, Merck Life Science, N7280, 1:1000) were conducted. Avidin–biotin complex method (ABC kit, Vector Laboratories, PK-6200, 1:200) was used for the antibodies visualization, according to the vendor’s instructions. The immunostained sections were mounted on microscope slides coated with gelatine, dehydrated, and coverslipped using DPX medium (Sigma Aldrich, 06522).

### Astrogliosis and microgliosis assessment

**Fig. 2:**
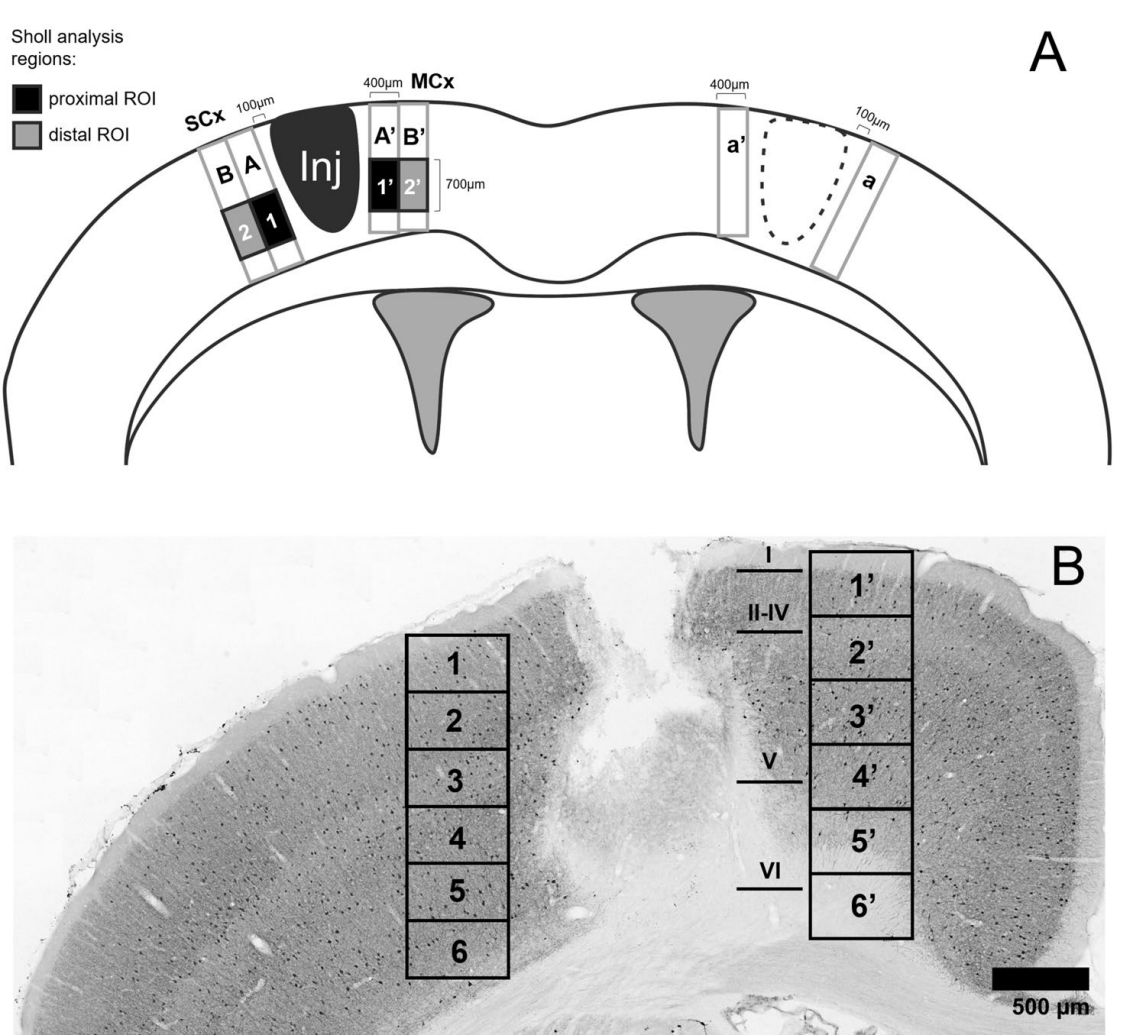
The regions chosen for the analysis of astrogliosis and microgliosis (**A**) and neuronal density (**B**). **A**—astrogliosis and microgliosis were analysed in the somatosensory cortex (SCx) and motor cortex (MCx), with a margin of 100 µm from the lesion border. Two subzones were delineated: proximal (100–500 µm from the lesion site; A, A’) and distal (500–900 µm from the lesion site; B, B’). In every one of these subzones, ROIs for Sholl analysis were destined: proximal ROIs (1, 1’) and distal ROIs (2, 2’). The contralateral reaction was assessed in two subzones in the right hemisphere, corresponding to proximal subzones around the injury (a, a’, respectively). **B**—the immunopositive neurons were counted in 6 depth levels of SCx (1–6) and MCx (1’−6’), and in corresponding areas in contralateral hemisphere

The neocortices were photographed using Capture software, Opta Tech Camera Mi20 and microscope Nikon ECLIPSE 200, under 10 × objective. Then, using Microsoft ICE software, they were merged into panoramic images of entire perilesional and contralateral cortical areas. These images were analysed in FIJI software. In the left cerebral hemisphere, two zones of interest were delineated through the whole thickness of the cerebral cortex, perpendicularly to its surface, laterally and medially from the lesion cavity. They represented the somatosensory (SCx) and motor cortices (MCx), respectively. These zones (each of 800 µm width), located 100 µm from the lesion border, were divided perpendicularly into two equal subzones, proximal and distal from the lesion site, each of 400 µm width. Additionally, within each of the four subzones, a ROI (region of interest) was destined for measurements of glial morphology (Sholl analysis). In the contralateral, uninjured hemisphere, the areas corresponding to proximal subzones around the injury site were assigned. Figure 2A shows spatial relationships between all the above zones and the cerebral hemisphere. Corresponding regions were analysed in the left and right hemispheres of control, non-injured brains.

### Immunoreactive GFAP + area fraction measurement

The GFAP + area fraction was assessed in every chosen neocortical subzone. Briefly, the image was split into channels, and the red and blue channels were discarded. In the green channel the background was reduced (Subtract Background, rolling = 50 light), the contrast was locally enhanced with the CLAHE algorithm implemented in FIJI (blocksize = 70, histogram = 256, maximum = 3) and images were thresholded (0, 170) and GFAP + area fraction was measured in binary images.

### Immunoreactive Iba1 + area fraction measurement

The Iba1 + area fraction was assessed in neocortical subzones in the same way as for the GFAP. The green channel was used for the analysis, the background was reduced (Subtract Background, rolling = 50 light), the contrast was locally enhanced with the CLAHE algorithm implemented in FIJI (blocksize = 69, histogram = 256, maximum = 3) and images were thresholded (0, 200) and Iba1 + area fraction was measured in binary images.

### Morphological analysis of astrocytes and microglia

The morphological analysis of astrocytes and microglia was performed for the cells creating glial scar around the lesion cavity in the left hemisphere of injured males and females. Four rectangular ROIs, 400 × 700 µm each, were selected for every animal, within the abovementioned subzones in the somatosensory and motor cortex, 100–500 µm (proximal ROI) and 500–900 µm (distal ROI) from the lesion cavity. Figure 2A presents the location of the chosen ROIs.

One to three astrocytes and microglia from each ROI were photographed under 100 × magnification using the EDF function of Capture software, enabling the collection of the data from the whole depth of the slice. Then, the green channel of the images was processed in FIJI. For cortical astrocytes, the images were adjusted (Subtract Background, rolling = 150 light; Unsharp Mask: radius = 9 mask = 0.60, Median: radius = 9; Enhance Contrast: saturated = 5) and images were thresholded (0, 233). Then, to obtain a complete silhouette, each image was manually edited under the view of the original image of the cell, to avoid bias. Some pixels were added or removed to separate the cell’s ramifications from neighbouring cells or join separated processes to the selected cell. This method has already been successfully implemented in glial cells morphological analysis (Fernández-Arjona et al. 2017). Similar image processing was performed for microglia, with subsequent parameters: Subtract Background, rolling = 300 light; Unsharp Mask: radius = 9 mask = 0.60; Median: radius = 8; Enhance Contrast: saturated = 5, threshold (0, 225).

The morphometric analysis was performed in binary images using the following options and plugins in FIJI: Neuroanatomy (Legacy: Sholl analysis (from image) option), Set measurements, and FracLac. The Sholl analysis was performed with concentric circles of 1 µm radius steps, starting 4 µm for astrocytes and 6 µm for microglia from the centre of the soma, which was determined by the average size of the investigated cells’ somas. The remaining parameters were left at their default settings. The number of primary branches was assessed visually. The following parameters were used from the Set measurements option: *Cell area*, *Cell perimeter*, *Cell circularity*. The fractal analysis was performed according to the protocol of Fernández-Arjona et al. (2017), with *Fractal dimension* *(D)* measured on the outlined cell’s silhouette and the following parameters measured on the filled cell’s silhouette: *Convex hull area (CHA)*, *Convex hull perimeter (CHP)*, *Convex hull circularity (CHC)*, *Cell density*, *Convex hull span ratio (Span ratio), Maximum Span Across the Convex Hull (MSACH), Diameter of the bounding circle (BC diameter),* the ratio maximum/minimum convex hull radii *(Max Min radii),* the mean radius *(Mean radius),* and *Lacunarity.* Table 2 presents the analysed morphological parameters and their description.

**Table 2:** The parameters measured in astrocytes and microglia during morphological analysis

Parameter	Description
Cell area [µm^2^]	Total number of pixels present in the filled shape of the cell image, transformed to µm^2^
Cell perimeter [µm]	The sum of pixels present in the single outline cell shape and in the internal holes, transformed to µm; counted manually
Cell circularity	4π*Area/Perimeter^2^
Length of processes [µm]	The length of the longest cell’s process, in µm (Ending radius estimated by Sholl Analysis)
Sum of intersections (Sum inters)	The sum of all intersections
Mean of intersections (Mean inters)	Sum inters divided by the number of intersecting radii
Median of intersections (Median inters)	The median value of sampled intersections
Radius of highest count of intersections (Max inters radius)	The distance at which the highest count of intersections occurred
Ramification index (Ramif ind)	The ratio of Max inters (maximum value of intersections with a single circle, estimated by Sholl analysis) and primary branches; counted manually
Critical value	The local maximum of the polynomial fit (number of intersections at Critical radius)
Critical radius	The distance at which Critical value occurs
Convex hull area (CHA) [px^2^]	The area of the smallest convex polygon containing the whole cell shape
Cell density	Cell Area/CHA
Convex hull perimeter (CHP) [px]	The perimeter of the smallest convex polygon containing the whole cell shape
Convex hull circularity (CHC)	4π*CHA/CHP^2^
Convex hull span ratio (Span ratio)	The ratio of the major to the minor axes of the convex hull
Maximum span across the convex hull (MSACH) [px]	The maximum distance between two points across the convex hull
The ratio maximum/minimum convex hull radii (Max Min radii)	The division of the largest to the smallest radius from the centre of mass of the convex hull to an exterior point
Diameter of the bounding circle (BC diameter) [px]	The diameter of the smallest circle that encloses the convex hull
Mean radius [px]	The mean length from the centre of mass of the convex hull to an exterior point
Lacunarity	The measure of cell’s *gappiness* and *heterogeneity.* The mean lacunarity for the image was calculated by FracLac as described by Karperien et al. (2011)
Fractal dimension (D)	The measure of cell’s complexity. The mean box counting dimension for the image was calculated by FracLac as described in https://imagej.net/ij/plugins/fraclac/FLHelp/Glossary.htm#meandb

### Neuronal loss assessment in the cortex

The numbers of parvalbumin-expressing (PV +), neuropeptide Y-expressing (NPY +) and nNOS-expressing (nNOS +) neurons were assessed separately for the perilesional motor and somatosensory cortices. The examinations were performed as reported previously (Setkowicz et al. 2016). Briefly, the cells were counted manually using an eyepiece with a square 10 × 10 mm frame which under a 400 × magnification delimited a 250 × 250 µm unit area of the observed tissue. Neurons were counted on both sides of the injury, with a margin of one frame (250 µm) from injury, within stripes extending over the whole thickness of the cerebral hemisphere. The width of the examined tissue stripe was 500 μm, which was equal to twice the width of the frame used during the counting. The whole thickness of each cerebral hemisphere wall was divided into six equal levels, and the recorded neurons were assigned to respective depth levels according to their locations. Figure 2B presents the spatial relations between the depth levels and the cortical layers and underlying white matter. The corresponding regions were analysed in the contralateral hemisphere and in the control brains.

### Statistical analysis

The statistical analysis was performed in R software. The outliers were detected with Grubb’s test. The heterogeneity of variance (Levene’s test), the non-normal distribution of data values (Shapiro–Wilk test) and the small and variable numbers of animals in the examined groups excluded ANOVA application. The differences between groups were tested by the non-parametric Wilcoxon rank-sum test (Mann–Whitney test). The level of statistical significance was set at 0.05. The percentage differences between groups are presented as percentage differences between medians. For PCA analysis, the original data, before outliers elimination, were used, in order to avoid replacing the lacking examples with mean values. PCA was carried out using the princomp function (R software). Analysis was performed on 9 selected parameters. We rejected parameters based on which other parameters were calculated (such as cell area, perimeter, maximum number of intersections), and parameters for which the effect size (Cohen's d) was small or negligible. Two first principal components together explained about 85% of observed variability for GFAP-positive cells.

## Results

### Reactive astrogliosis in perilesional somatosensory cortex

**Fig. 3:**
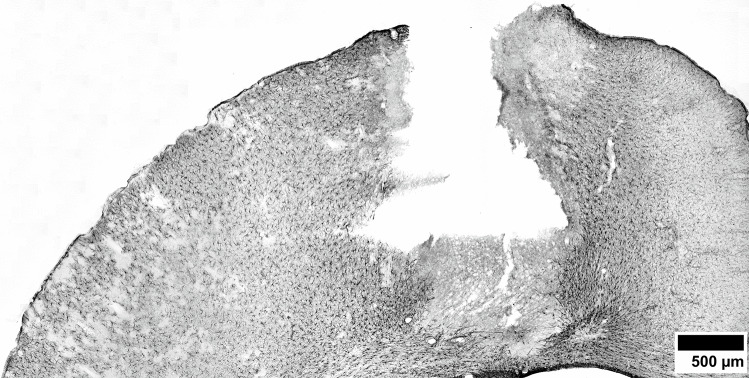
Location of the lesion site in the left cerebral hemisphere. Male rat, 2 days after TBI, GFAP immunostaining for revealing the perilesional astrogliosis

As a result of penetrating TBI, a focal lesion with a clearly visible lesion core was produced (Fig. 3).

**Fig. 4:**
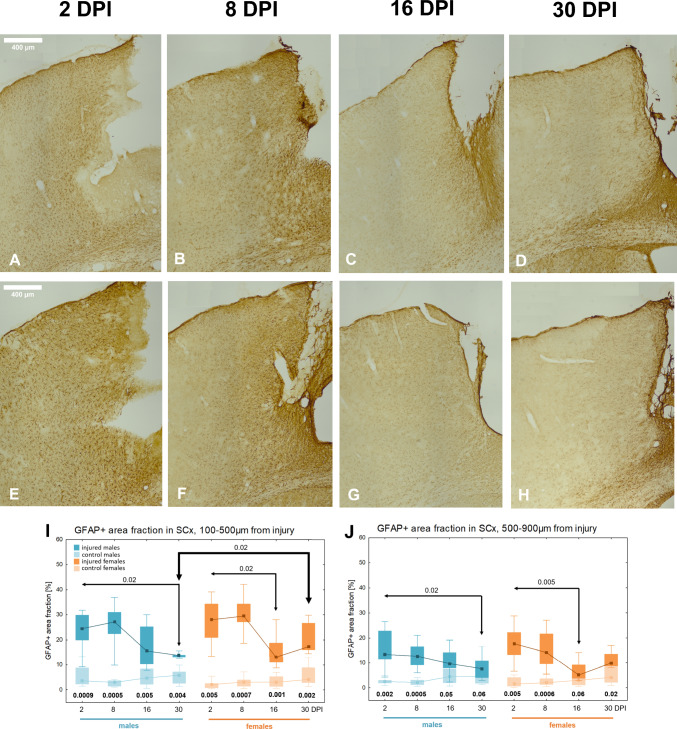
Glial scar development in SCx after penetrating brain injury, GFAP immunoreactivity. Representative images of GFAP-immunoreactive astrocytes in injured somatosensory cortex at 2, 8, 16, and 30 days post-injury in females (**A**–**D**) and males (**E**–**H**). Variations of GFAP + area fraction in injured and control animals in SCx: the proximal (**I**) and distal subzone (**J**). Each box and whisker graph shows the median (small square in the box), the 25–75% variability range (large box), maximal and minimal values (whiskers). Decimal indexes in bold show levels of statistical significance of differences between injured and control groups. Decimal indexes with light arrows show levels of statistical significance of differences between injured age groups within sexes. Decimal indexes with bolded arrows show levels of statistical significance of differences between injured males and females

In the surrounding tissue, robust reactive astrogliosis was observed, manifested by an increase in GFAP immunoreactivity at 2DPI, which persisted until 30DPI. Figure 4 illustrates the development of the glial scar in both females and males over 30 days following injury. At 2DPI, hypertrophic astrocytes were evident around the site of injury. By 30DPI, GFAP immunoreactivity decreased, and the lesion was separated from the surrounding tissue by a well-defined glial scar, formed by astrocytic processes (Fig. 4A–H). GFAP + area fraction in the proximal subzone (100-500 µm from injury) was significantly higher in injured groups compared to controls at all examined time points in both sexes. A significant decrease in the GFAP + area fraction was observed between 2 and 16DPI (p < 0.02) in females, and between 2 and 30DPI (p < 0.02) in males, indicating a gradual reduction of astrogliosis and a decline in GFAP level (Fig. 4I). In the distal subzone (500-900 µm from injury), GFAP + area fraction was also increased relative to controls but declined over time, reaching levels comparable to controls at 16DPI (p = 0.05) and 30DPI (p = 0.06) in males, and at 16DPI (p = 0.06) in females. A significant decrease in the GFAP + area fraction was observed between 2 and 16DPI (p < 0.005) in females, and between 2 and 30DPI (p < 0.02) in males (Fig. 4J). Notably, at 30DPI, females exhibited a higher GFAP + area fraction in the proximal subzone compared to males (p < 0.02), indicating stronger astrocyte activation in the proximity of injury in females (Fig. 4I).

### Reactive astrogliosis in perilesional motor cortex

**Fig. 5:**
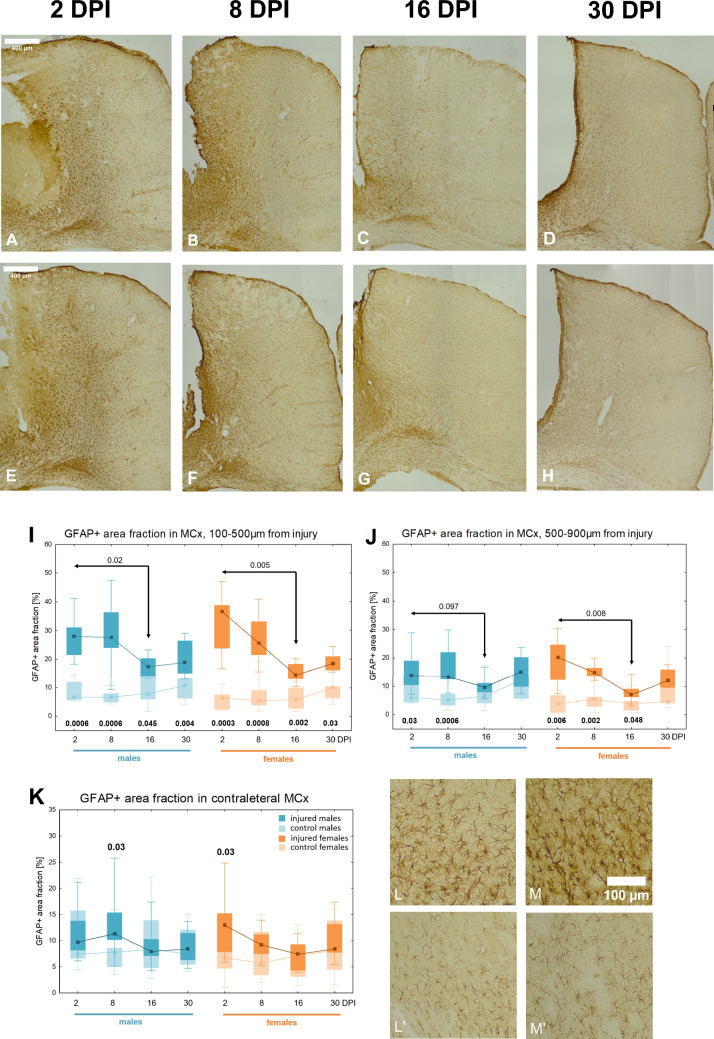
GFAP immunoreactivity in perilesional and contralateral MCx after penetrating brain injury. Representative images of GFAP-immunoreactive astrocytes in injured motor cortex at 2, 8, 16, and 30 days post-injury in females (**A**–**D**) and males (**E**–**H**). Variations of GFAP + area fraction in injured and control animals in MCx: the proximal (**I**) and distal subzone (**J**). Variations of GFAP + area fraction in injured and control animals in contralateral MCx (**K**). Representative images of GFAP-immunoreactive astrocytes in contralateral motor cortex (**L**–**M**): L-injured males 8DPI, L’—control males P38. M-injured females 2DPI, M’-control females P32. Each box and whisker graph shows the median (small square in the box), the 25–75% variability range (large box), maximal and minimal values (whiskers). Decimal indexes in bold show levels of statistical significance of differences between injured and control groups. Decimal indexes with light arrows show levels of statistical significance of differences between injured age groups within sexes

Similar patterns were observed in the motor cortex (Fig. 5A-H). The GFAP + area fraction in the proximal subzone was significantly elevated compared to the control groups at each time point, in both females and males. A significant decrease in the GFAP + area fraction was observed in both sexes between 2 and 16DPI (p < 0.005 for females, p < 0.02 for males) (Fig. 5I). In the distal subzone, GFAP + area fraction was also increased relative to controls. In males, this increase was significant only at 2DPI (p < 0.03) and 8DPI (p < 0.0006), whereas in females, elevated GFAP + area fraction persisted at 2DPI (p < 0.006), 8DPI (p < 0.002), and 16DPI (p < 0.05). Females exhibited a significant decrease in GFAP + area fraction between 2 and 16DPI (p < 0.008), with a similar but non-significant trend observed in males (p < 0.1) (Fig. 5J). No sex differences were observed in the GFAP + area fraction in the motor cortex.

### Contralateral reaction of astrocytes in motor and somatosensory cortex

The contralateral reaction of astrocytes, assessed in the motor cortex of the right hemisphere of injured animals compared to control groups, was observed at 2DPI (p < 0.03) in females and at 8DPI (p < 0.03) in males (Fig. 5K–M). In the somatosensory cortex, no contralateral astrocyte response was observed (data not shown).

### Reactive microgliosis in perilesional somatosensory cortex

**Fig. 6:**
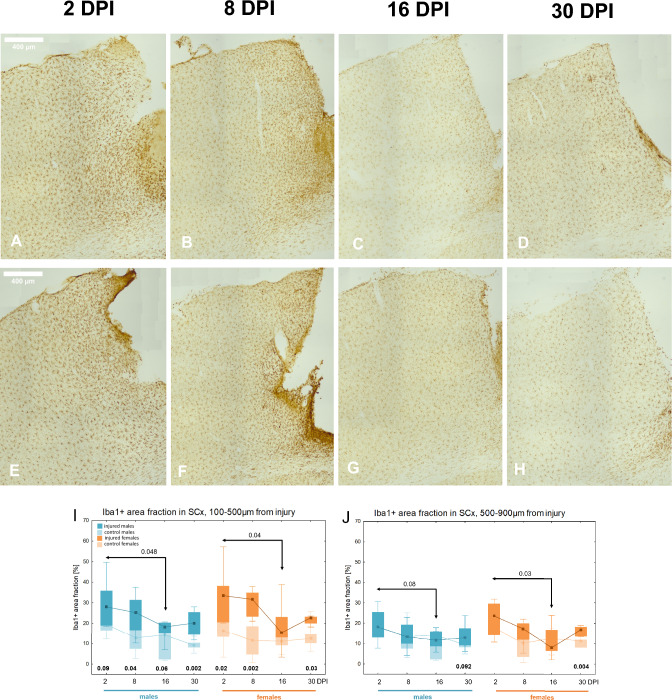
Microgliosis in perilesional SCx after penetrating brain injury, Iba1 immunoreactivity. Representative images of Iba1-immunoreactive microglia in injured somatosensory cortex at 2, 8, 16, and 30 days post-injury in females (**A**–**D**) and males (**E**–**H**). Variations of Iba1 + area fraction in injured and control animals in SCx: the proximal (**I**) and distal subzone (**J**). Each box and whisker graph shows the median (small square in the box), the 25–75% variability range (large box), maximal and minimal values (whiskers). Decimal indexes in bold show levels of statistical significance of differences between injured and control groups. Decimal indexes with light arrows show levels of statistical significance of differences between injured age groups within sexes

Microglial cells are the first to respond to tissue damage, undergoing structural and functional changes aimed at restoring homeostasis. In order to determine the microglial response to TBI, the area occupied by these cells was analysed over 30 days following injury. Figure 6A–H illustrates the progression of microglial response in females and males. At 2 days post injury, hypertrophic microglial cells are evident, accumulating around the lesion site and gradually adopting a more ramified morphology by 16DPI and 30DPI.

The Iba1 + area fraction tended to reach higher values compared to the control groups, reaching statistical significance at 2DPI (p < 0.02), 8DPI (p < 0.002), and 30DPI (p < 0.03) in females, and at 8DPI (p < 0.04) and 30DPI (p < 0.002) in males, indicating pronounced microgliosis in the proximal subzone. A significant decrease in the Iba1 + area fraction occurred between 2 and 16DPI in both females (p < 0.04) and males (p < 0.05) (Fig. 6I). In the distal subzone, the Iba1 + area fraction was significantly higher in females at 30DPI compared to the control group (p < 0.004), with a similar trend observed in males (p < 0.1), suggesting a prolonged microglial activation. Females exhibited a significant reduction in Iba1 + area fraction between 2 and 16DPI (p < 0.03), with a comparable but non-significant trend in males (p < 0.08) (Fig. 6J). No significant sex differences were observed in the Iba1 + area fraction.

### Reactive microgliosis in perilesional motor cortex

**Fig. 7:**
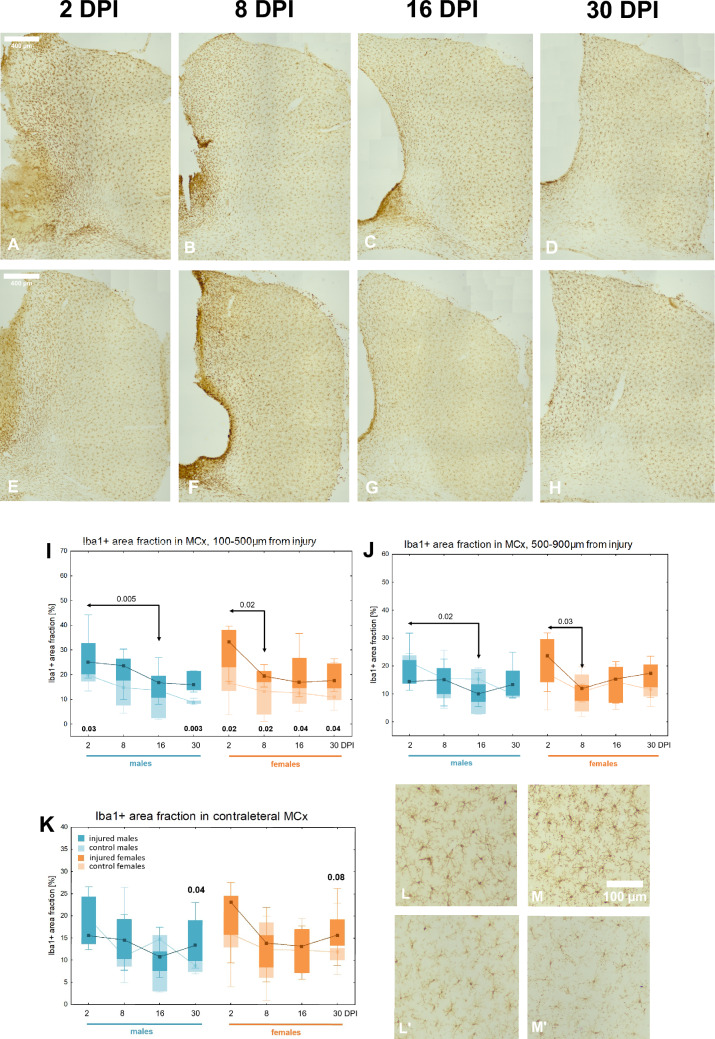
Microgliosis after penetrating brain injury in perilesional and contralateral MCx. Representative images of Iba1-immunoreactive microglia in injured motor cortex at 2, 8, 16, and 30 days post-injury in females (**A**–**D**) and males (**E**–**H**). Variations of Iba1 + area fraction in injured and control animals in MCx: the proximal (**I**) and distal subzone (**J**). Variations of Iba1 + area fraction in injured and control animals in contralateral MCx (**K**). Representative images of Iba1-immunoreactive microglia in contralateral motor cortex (**L**–**M**): L-injured males 30DPI, L’—control males P60. M-injured females 30DPI, M’-control females P60. Each box and whisker graph shows the median (small square in the box), the 25–75% variability range (large box), maximal and minimal values (whiskers). Decimal indexes in bold show levels of statistical significance of differences between injured and control groups. Decimal indexes with light arrows show levels of statistical significance of differences between injured age groups within sexes

Figure 7A-H illustrates the course of microglial response in the motor cortex. The Iba1 + area fraction in proximal subzone tended to reach higher values compared to the control groups, reaching statistical significance at 2DPI (p < 0.02), 8DPI (p < 0.02), 16DPI (p < 0.04), and 30DPI (p < 0.04) in females, and at 2DPI (p < 0.03) and 30DPI (p < 0.003) in males. A significant decrease in Iba1 + area fraction occurred between 2 and 8DPI (p < 0.02) in females, and between 2 and 16DPI (p < 0.005) in males (Fig. 7I). The Iba1 + area fraction in the distal subzone did not differ significantly from the area fraction in the control groups. A significant decrease in the Iba1 + area fraction occurred between 2 and 8DPI (p < 0.03) in females, and between 2 and 16DPI (p < 0.02) in males (Fig. 7J). No sex differences were observed in the Iba1 + area fraction.

### Contralateral reaction of microglia in motor and somatosensory cortex

The contralateral reaction of microglia, assessed in the motor cortex of the right hemisphere of injured animals compared to control groups, was observed at 30DPI (p < 0.04) in males, and a similar trend was noted at 30DPI (p < 0.08) in females (Fig. 7K-M). In the somatosensory cortex, no contralateral microglial response was observed (data not shown).

### Morphological changes of astrocytes in perilesional somatosensory cortex

**Fig. 8:**
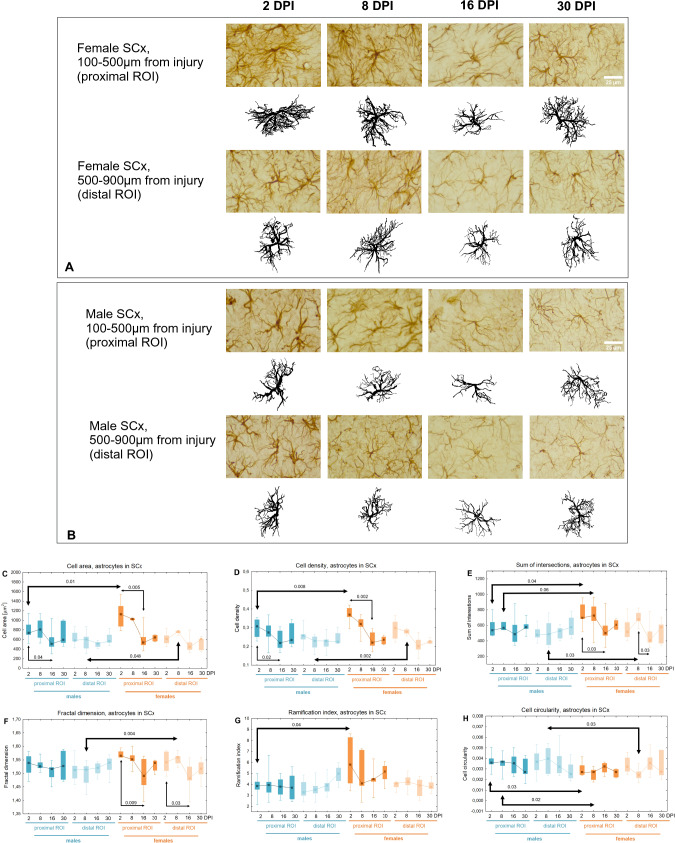
Representative reactive astrocytes in injured somatosensory cortex of females (**A**) and males (**B**). **C**–**H**: shape related parameters in reactive astrocytes in proximal and distal ROI of injured somatosensory cortex. Each box and whisker graph shows the median (small square in the box), the 25–75% variability range (large box), maximal and minimal values (whiskers). Decimal indexes with light arrows show levels of statistical significance of differences between injured age groups within sexes. Decimal indexes with bolded arrows show levels of statistical significance of differences between injured males and females

**Fig. 9:**
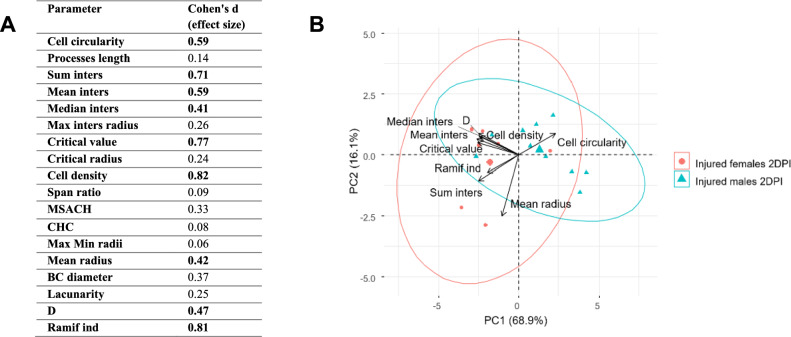
PCA results for morphological parameters of astrocytes in proximal ROI of somatosensory cortex in injured males and females 2DPI. **A**—effect size of different morphological parameters of astrocytes. Parameters in bold were chosen for PCA. **B**—distribution of male and female astrocytes on the principal components plane. Ellipses show 95% confidence interval per each group. Arrows show loadings obtained for each parameters (indicate the contribution of original variables in creating new components)

To further investigate the glial response to brain injury, a morphological analysis of astrocytes was performed in the perilesional somatosensory cortex, both closer to the injury (proximal ROI) and farther away from the injury (distal ROI). Some of the studied parameters describing cell shape showed changes over the course of one month post-injury and varied depending on sex. Figure 8 presents the representative cells in each age group for females and males. A downward tendency was observed in proximal ROI of both females and males for the *Cell area* and *Cell density* (Fig. 8C–D). The *Sum of intersections* remained constant in males. In females, within the proximal ROI, it reached the highest values at 2 and 8DPI and decreased by 29% between 2 and 16DPI (p < 0.03), while in the distal ROI, the highest value was observed at 8 DPI, followed by a significant decrease by 25% to 16DPI (p < 0.03) (Fig. 8E). The *Fractal dimension* remained constant in males. In females, both in the proximal and distal ROIs, it significantly decreased between 2 and 16DPI (p < 0.009, 5% for proximal ROI, p < 0.03, 4% for distal ROI) (Fig. 8F). Sex differences were observed at 2 and 8 DPI. *Cell Area* (proximal ROI: 2DPI, p < 0.01, 53%; distal ROI: 8DPI, p < 0.05, 27%), *Cell Density* (proximal ROI: 2DPI, p < 0.008, 20%; distal ROI: 8DPI, p < 0.002, 21%), *Sum of Intersections* (proximal ROI: 2DPI, p < 0.04, 30%, 8DPI, p < 0.06, 28%; distal ROI: 8DPI, p < 0.03, 39%), *Fractal Dimension* (distal ROI 8DPI, p < 0.004, 3%), and *Ramification Index* (proximal ROI: 2DPI, p < 0.04, 51%) were higher in females than in males (Fig. 8C–G). *Cell Circularity* was lower in females compared to males (proximal ROI: 2DPI p < 0.03, 25%, 8DPI p < 0.02, 26%; distal ROI 8DPI p < 0.03, 40%) (Fig. 8H). These differences suggest a more hypertrophic and complex morphology of astrocytes in females during the initial days following TBI. Figure 9 presents the PCA results for astrocytes from females and males at 2DPI in the proximal ROI. The first principal component explained approximately 70% of the observed variability and was negatively correlated with parameters regarding the cell complexity. The female group shift to the left in relation to the centre of the coordinate system indicates that females have more complex cell morphology.

### Morphological changes of astrocytes in perilesional motor cortex

**Fig. 10:**
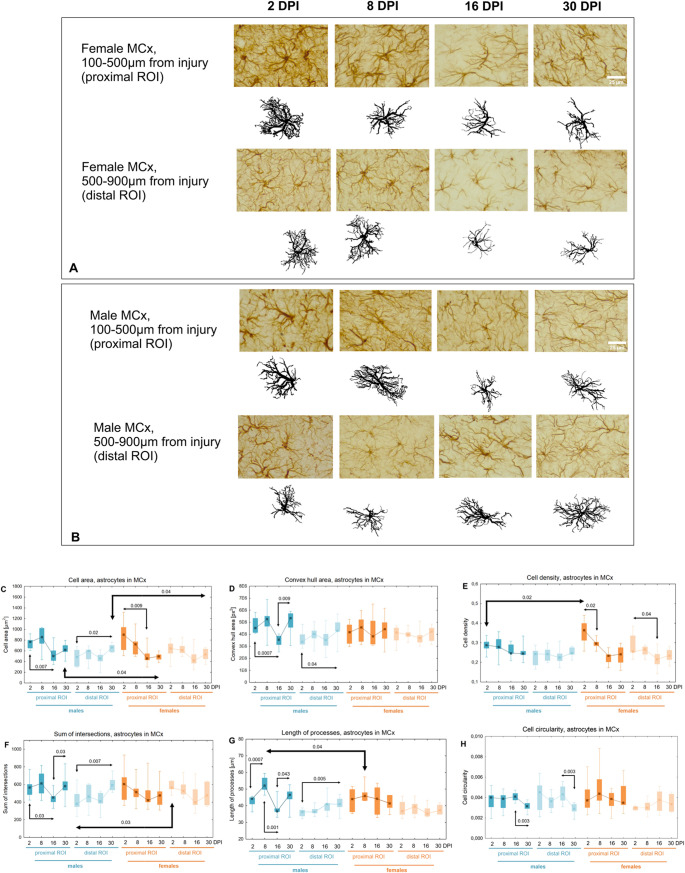
Representative reactive astrocytes in injured motor cortex of females (**A**) and males (**B**). **C-H**: shape related parameters in reactive astrocytes in proximal and distal ROI of injured motor cortex. Each box and whisker graph shows the median (small square in the box), the 25–75% variability range (large box), maximal and minimal values (whiskers). Decimal indexes with light arrows show levels of statistical significance of differences between injured age groups within sexes. Decimal indexes with bolded arrows show levels of statistical significance of differences between injured males and females

Similar trends were observed in the motor cortex. Figure 10 presents representative cells from each age group for females and males. Both female and male astrocytes in proximal ROI exhibited a downward trend in *Cell area.* In males, *Cell area* in the distal ROI increased by 27% between 2 and 30DPI (p < 0.02) (Fig. 10C). The *Convex hull area* remained constant in females. In males, it decreased by 21% between 2 and 16DPI in the proximal ROI (p < 0.0007), and then increased by 50% between 16 and 30DPI (p < 0.009). In the distal ROI, it significantly increased by 27% between 2 and 30DPI (p < 0.04) (Fig. 10D). *Cell density* did not change in males. In females, it was highest in the proximal ROI at 2DPI, then significantly decreased by 19% between 2 and 8DPI (p < 0.02), and in the distal ROI between 2 and 16DPI (p < 0.04, 15%) (Fig. 10E). The *Sum of intersections* remained constant in females; in males, it significantly decreased by 20% between 2 and 16DPI in the proximal ROI (p < 0.03), then increased by 28% between 16 and 30DPI (p < 0.03). In the distal ROI, it increased by 35% between 2 and 30DPI (p < 0.007) (Fig. 10F). The *Length of processes* remained constant in females. In males, the *Length of processes* in the proximal ROI increased by 18% between 2 and 8DPI (p < 0.0007), decreased by 30% between 8 and 16DPI (p < 0.001), and then increased by 27% between 16 and 30DPI (p < 0.05). In the distal ROI, the Length of processes increased by 13% between 2 and 30DPI (p < 0.005) (Fig. 10G).

*Cell circularity* significantly decreased in males in both proximal and distal ROIs between 16 and 30DPI (p < 0.003, 24% in proximal ROI; p < 0.003, 32% in distal ROI) (Fig.10H). *Cell density* was 27% higher in females than in males at 2DPI in the proximal ROI (p < 0.02) (Fig. 10E). The *Sum of intersections* was 48% higher in females than in males at 2DPI in the distal ROI (p < 0.03) (Fig. 10F). *Cell area* was significantly higher in males than in females at 30DPI in both ROIs (p < 0.04, 24% in both ROIs) (Fig. 10C). The *Length of processes* was 14% higher in the proximal ROI at 8DPI in males than in females (p < 0.04) (Fig. 10G).

### Morphological changes of microglia in perilesional somatosensory cortex

**Fig. 11:**
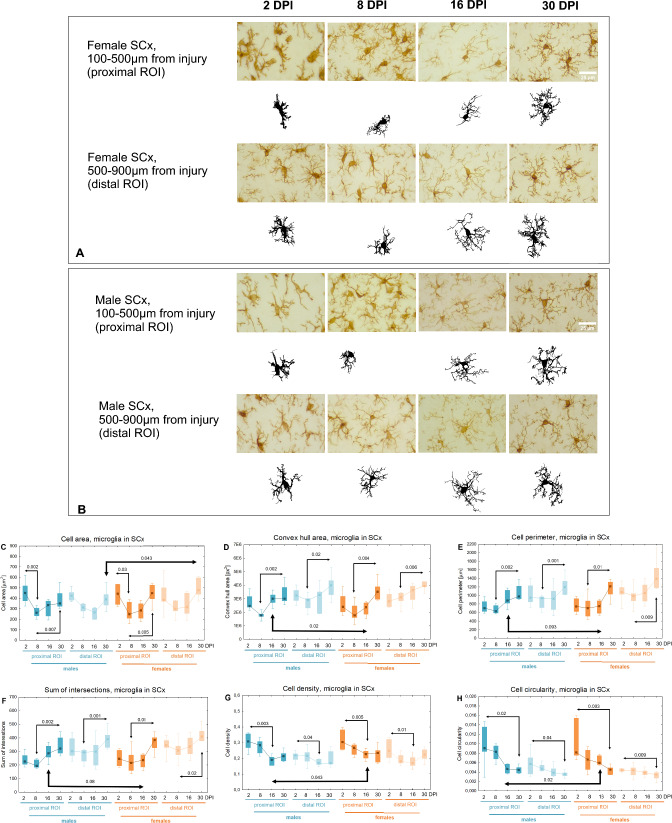
Representative reactive microglia in injured somatosensory cortex of females (**A**) and males (**B**). **C–H**: shape related parameters in reactive microglia in proximal and distal ROI of injured somatosensory cortex. Each box and whisker graph shows the median (small square in the box), the 25–75% variability range (large box), maximal and minimal values (whiskers). Decimal indexes with light arrows show levels of statistical significance of differences between injured age groups within sexes. Decimal indexes with bolded arrows show levels of statistical significance of differences between injured males and females

Similar trends in the morphological changes of microglia over the month following injury were observed in both sexes. Figure 11 presents representative cells from each age group for females and males. *Cell area* in the proximal ROI significantly decreased between 2 and 8DPI (p < 0.03, 44% for females, p < 0.002, 42% for males), and then increased between 8 and 30DPI (p < 0.005, 80% for females, p < 0.007, 35% for males) (Fig. 11C). An upward trend was observed for parameters such as *Convex hull area*, *Cell perimeter*, and *Sum of intersections*, while a downward trend was noted for *Cell density* and *Cell circularity*, indicating a gradual increase in cell complexity (Fig. 11D–H). At 16DPI in the proximal ROI, females exhibited lower *Convex hull area* (p < 0.02, 20%), *Cell perimeter* (p < 0.1, 15%), and *Sum of intersections* (p < 0.08, 17%), as well as higher *Cell density* (p < 0.05, 18%) and *Cell circularity* (p < 0.02, 28%) compared to males, suggesting a less complex morphology in females at this time point (Fig. 11D-H). At 30DPI in the distal ROI, females had 28% larger *Cell area* than males (p < 0.05) (Fig. 11C).

### Morphological changes of microglia in perilesional motor cortex

**Fig. 12:**
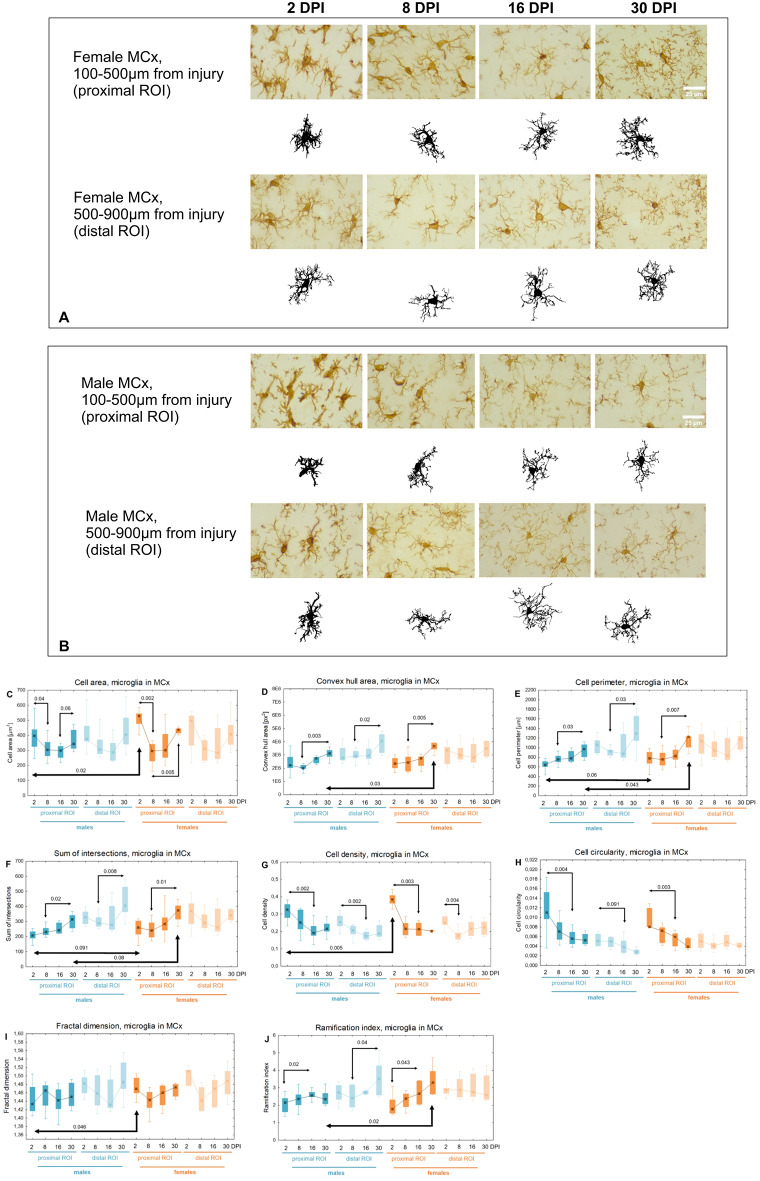
Representative reactive microglia in injured motor cortex of females (**A**) and males (**B**). **C**–**J**: shape related parameters in reactive microglia in proximal and distal ROI of injured somatosensory cortex. Each box and whisker graph shows the median (small square in the box), the 25–75% variability range (large box), maximal and minimal values (whiskers). Decimal indexes with light arrows show levels of statistical significance of differences between injured age groups within sexes. Decimal indexes with bolded arrows show levels of statistical significance of differences between injured males and females

The microglia in motor cortex underwent similar modifications. Figure 12 presents representative cells from each age group for females and males. *Cell area* in the proximal ROI significantly decreased between 2 and 8DPI (p < 0.002, 44% for females, p < 0.04, 23% for males), and then increased between 8 and 30DPI in females (p < 0.005, 47%) and between 16 and 30DPI in males (p < 0.06, 15%) (Fig. 12C). An upward trend was observed for parameters such as *Convex hull area*, *Cell perimeter*, *Sum of intersections*, and *Ramification index*, while a downward trend was noted for *Cell density* and *Cell circularity* (Fig. 12D-H, J). At 2DPI in the proximal ROI, females exhibited 33% larger *Cell area* (p < 0.02) and 18% higher *Cell density* (p < 0.005) compared to males, as well as 3% higher *Fractal dimension* (p < 0.05), with a similar trend in *Sum of intersections* (p < 0.1, 25%) and *Cell perimeter* (p < 0.06, 21%), suggesting greater cell complexity in females (Fig. 12C, G, I, F, E). At 30DPI in the proximal ROI, females had 18% larger *Convex hull area* (p < 0.03), 26% larger *Cell perimeter* (p < 0.05), and 41% higher *Ramification index* (p < 0.02) than males, with a similar trend in *Sum of intersections* (p < 0.08, 19%), indicating greater complexity and area occupied by cells in females (Fig. 12D, E, J, F).

### PV + neurons loss in somatosensory and motor cortex

**Fig. 13:**
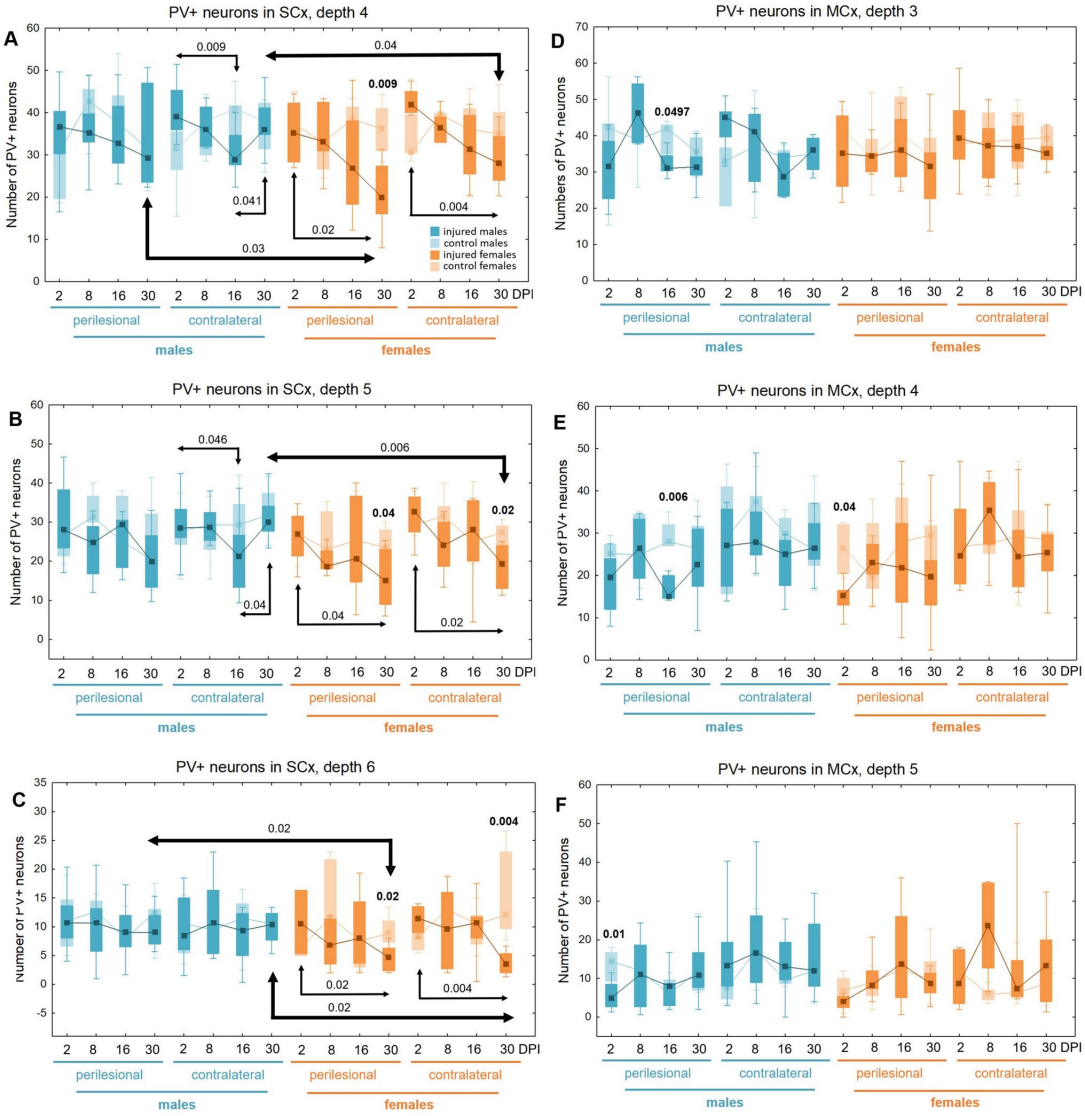
PV + neurons density in somatosensory cortex (**A**–**C**) and motor cortex (**D**–**F**) after TBI. Each box and whisker graph shows the median (small square in the box), the 25–75% variability range (large box), maximal and minimal values (whiskers). Decimal indexes in bold show levels of statistical significance of differences between injured and control group. Decimal indexes with light arrows show levels of statistical significance of differences between injured age groups within sexes. Decimal indexes with bolded arrows show levels of statistical significance of differences between injured males and females

A downward trend in the density of parvalbumin-expressing (PV +) neurons was observed in the somatosensory cortex around the injury site and in the contralateral area at depth levels 3–6, corresponding to layers V and VI of the cortex, in both females and males (Fig. 13). Injured females exhibited a substantial reduction in PV + neurons density 30 days following injury. The number of PV + neurons in the perilesional SCx of females was significantly reduced at 30DPI compared to 2DPI (levels 4, 5, 6: p < 0.02, p < 0.04, p < 0.02, respectively), the control group (levels 4, 5, 6: p < 0.009, p < 0.04, p < 0.02, respectively), as well as compared to males at 30DPI (levels 4, 6: p < 0.03, p < 0.02), indicating more pronounced loss of these cells in females. Similarly, there was a significant reduction of PV + neurons density in the contralateral SCx of females at 30DPI compared to 2DPI (levels 4, 5, 6: p < 0.004, p < 0.02, p < 0.004, respectively), the control group (levels 5, 6: p < 0.02, p < 0.004), and males at 30DPI (levels 4, 5, 6: p < 0.04, p < 0.006, p < 0.02) (Fig. 13A–C). A reduction in PV + neurons density in the perilesional cortex of injured males compared to the control group was observed at 16DPI in the motor cortex (MCx, levels 3, 4: p < 0.05, p < 0.006, respectively) (Fig. 13D–E). The number of PV + neurons in the contralateral SCx of injured males decreased at 16DPI compared to 2DPI (levels 4, 5: p < 0.009, p < 0.05), and then increased between 16 and 30DPI (levels 4, 5: p < 0.05, p < 0.04) (Fig. 13A–B). Both females and males exhibited an early reduction in PV + neurons density in the perilesional motor cortex compared to non-injured rats at 2DPI, followed by a recovery to levels comparable to the control group in further timepoints (MCx, level 4, p < 0.04 for females, MCx, level 5, p < 0.01 for males) (Fig. 13E–F). The changes in the density of PV + neurons after TBI across all cortical layers are presented in Tables 3 and 4.

**Table 3:**
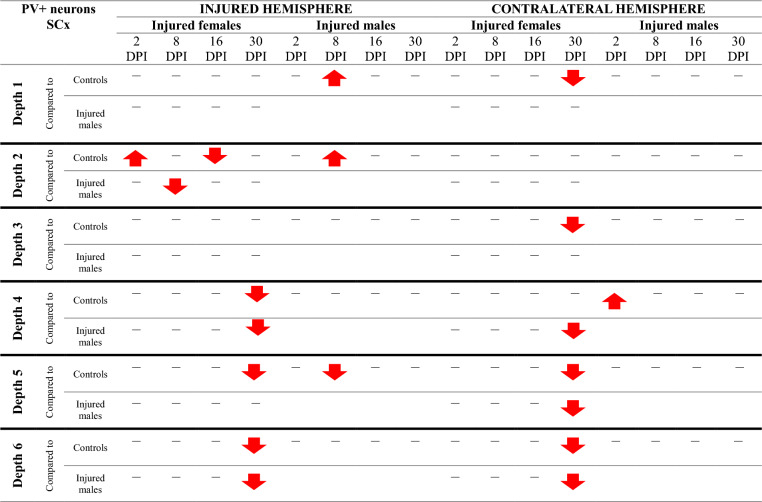
Changes of PV + neurons density after TBI in somatosensory cortex

The arrows indicate the increase or decrease of PV + neurons number at certain depth in injured males and females compared to controls (upper row of each depth section), or in injured females compared to injured males (lower row of each depth section)

**Table 4:**
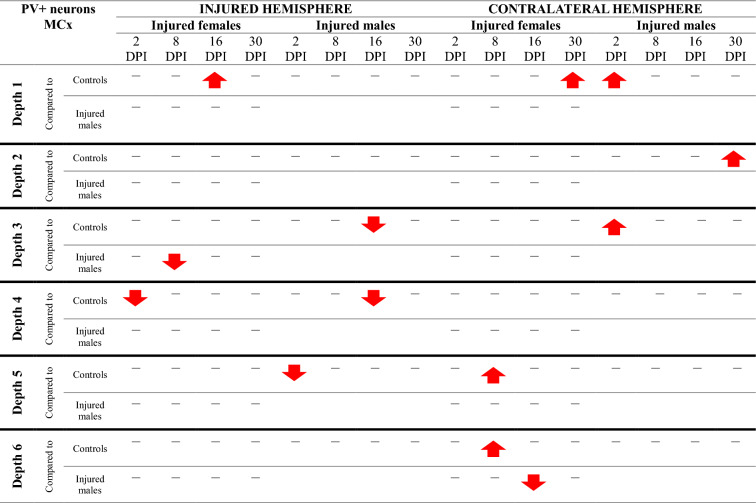
Changes of PV + neurons density after TBI in motor cortex

The arrows indicate the increase or decrease of PV + neurons number at certain depth in injured males and females compared to controls (upper row of each depth section), or in injured females compared to injured males (lower row of each depth section)

### NPY + neurons loss in somatosensory and motor cortex

**Fig. 14:**
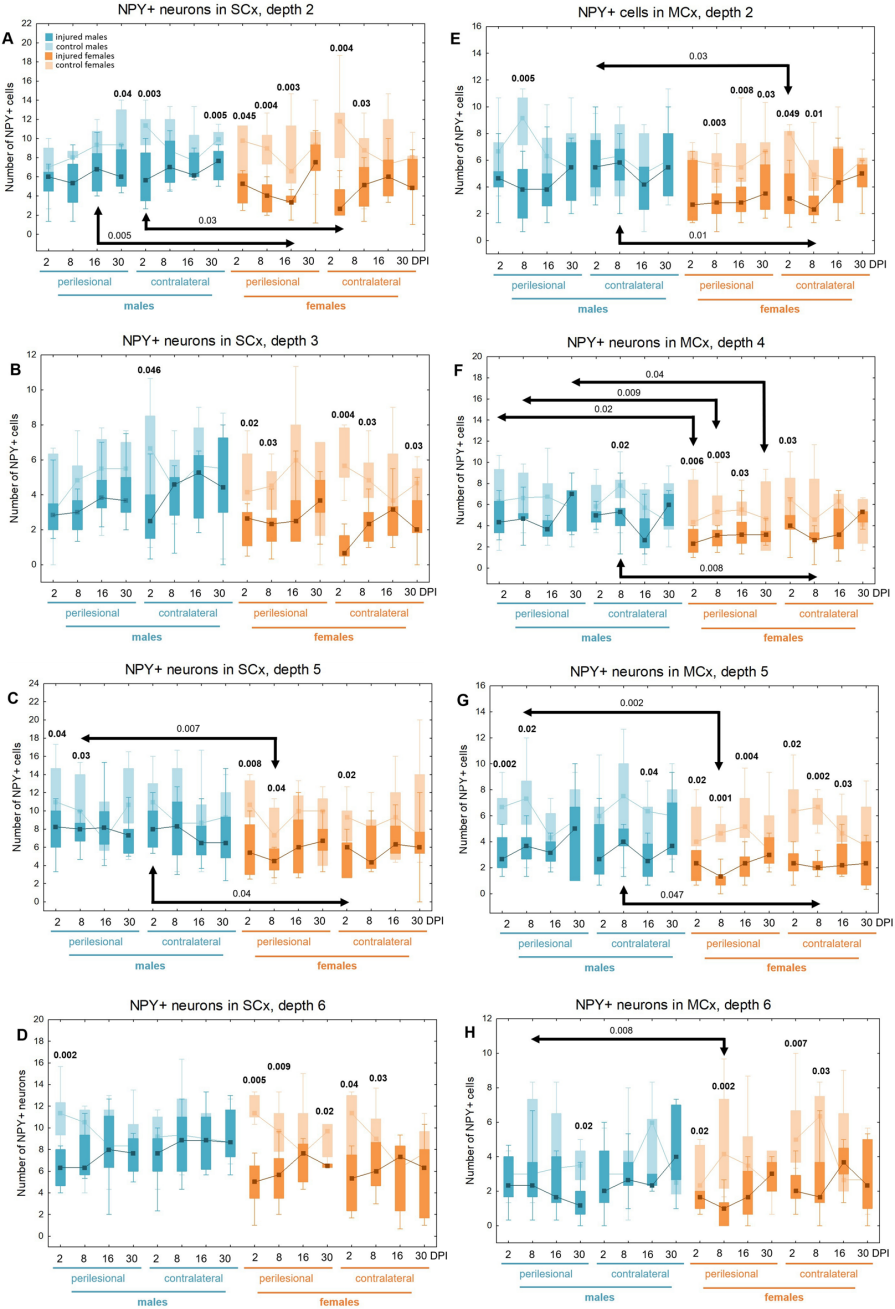
NPY + neurons density in somatosensory cortex (**A**–**D**) and motor cortex (**E**–**H**) after TBI. Each box and whisker graph shows the median (small square in the box), the 25–75% variability range (large box), maximal and minimal values (whiskers). Decimal indexes in bold show levels of statistical significance of differences between injured and control group. Decimal indexes with bolded arrows show levels of statistical significance of differences between injured males and females

Injured rats exhibited a reduction in NPY + neuron numbers across the full cortical depth, with pronounced decreases at levels 2, 4, 5, and 6 of the motor cortex, as well as at levels 2, 3, 5, and 6 of the somatosensory cortex (Fig. 14). Injured females exhibited significantly fewer NPY + neurons than controls at multiple time points. At 2DPI, reductions were observed in the motor cortex (MCx levels 4, 5, 6: p < 0.006, p < 0.02, p < 0.02, respectively) and somatosensory cortex (SCx levels 2, 3, 5, 6: p < 0.05, p < 0.02, p < 0.008, p < 0.005). Similar reductions were present at 8DPI (MCx levels 2, 4, 5, 6: p < 0.003, p < 0.003, p < 0.001, p < 0.002; SCx levels 2, 3, 5, 6: p < 0.004, p < 0.03, p < 0.04, p < 0.009), 16DPI (MCx levels 2, 4, 5: p < 0.008, p < 0.03, p < 0.004; SCx level 2: p < 0.003), and 30DPI (MCx level 2: p < 0.03; SCx level 6: p < 0.02) (Fig. 14A–H). A reduction in NPY + neuron numbers in injured males compared to controls was observed at 2DPI (MCx level 5, p < 0.002; SCx levels 5, 6: p < 0.04, p < 0.002, respectively), 8DPI (MCx levels 2, 5: p < 0.005, p < 0.02; SCx level 5: p < 0.03), and 30DPI (MCx level 6, p < 0.02; SCx level 2, p < 0.04) (Fig. 14A, C, D, E, G, H). At all examined time points, males exhibited significantly higher NPY + neuron densities in the perilesional cortex compared to females, with significant differences at 2DPI (MCx level 4, p < 0.02), 8DPI (MCx levels 4, 5, 6: p < 0.009, p < 0.002, p < 0.008 respectively; SCx level 5: p < 0.007), 16DPI (SCx level 2: p < 0.005), and 30DPI (MCx level 4: p < 0.04) (Fig. 14A, C, F–H).

**Fig. 15:**
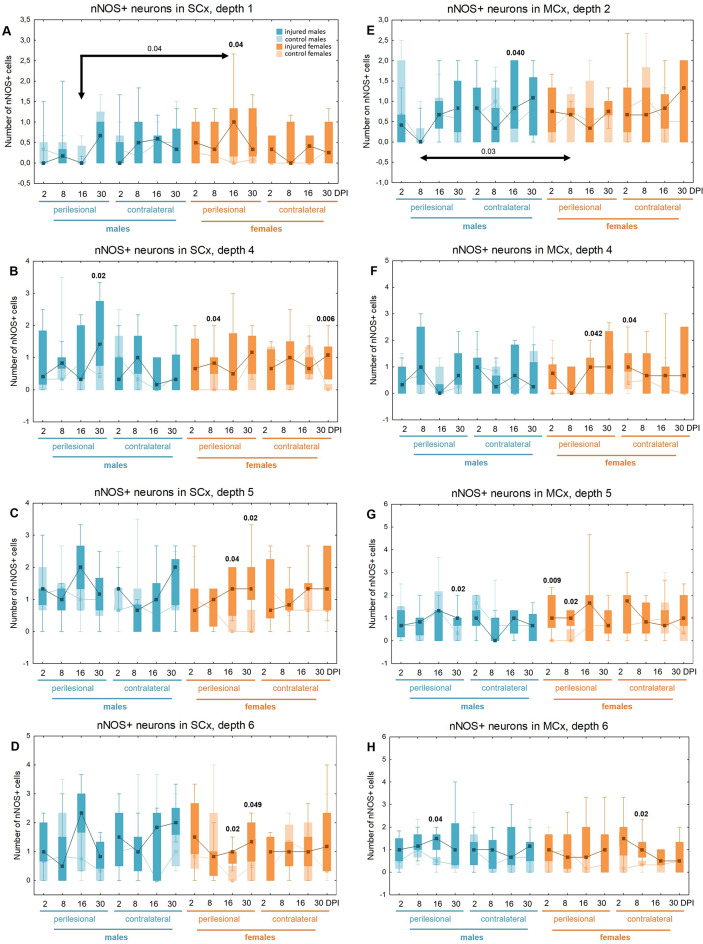
nNOS + neurons density in somatosensory cortex (**A**–**D**) and motor cortex (**E**–**H**) after TBI. Each box and whisker graph shows the median (small square in the box), the 25–75% variability range (large box), maximal and minimal values (whiskers). Decimal indexes in bold show levels of statistical significance of differences between injured and control group. Decimal indexes with bolded arrows show levels of statistical significance of differences between injured males and females

Contralateral reductions in NPY + neuron numbers relative to control levels were also observed. In females, these were detected at 2DPI (MCx levels 2, 4, 5, 6: p < 0.05, p < 0.03, p < 0.02, p < 0.007, respectively; SCx levels 2, 3, 5, 6: p < 0.004, p < 0.004, p < 0.02, p < 0.04), 8DPI (MCx levels 2, 5, 6: p < 0.01, p < 0.002, p < 0.03; SCx levels 2, 3, 6: p < 0.03, p < 0.03, p < 0.03), 16DPI (MCx level 5: p < 0.03), and 30DPI (SCx level 3: p < 0.03) (Fig. 14A–H). In males, contralateral reductions were identified at 2DPI (SCx levels 2, 3: p < 0.003, p < 0.05), 8DPI (MCx level 4: p < 0.02), 16DPI (MCx level 5: p < 0.04), and 30DPI (SCx level 2: p < 0.005) (Fig. 14A, B, F, G). Notably, males demonstrated significantly higher contralateral NPY + neuron densities compared to females at 2DPI (MCx level 2: p < 0.03; SCx levels 2, 5: p < 0.03, p < 0.04), and 8DPI (MCx levels 2, 4, 5: p < 0.01, p < 0.008, p < 0.05) (Fig. 15C, E–G). The changes in the density of NPY + neurons after TBI across all cortical layers are presented in Tables 5 and 6.

**Table 5:**
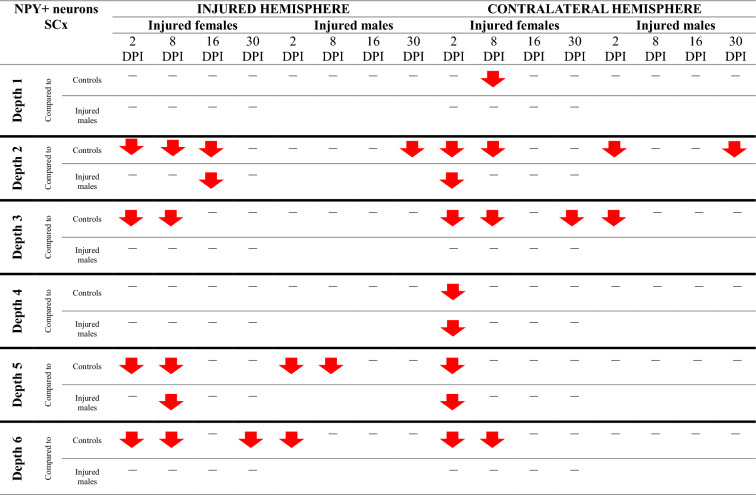
Changes of NPY + neurons density after TBI in somatosensory cortex

The arrows indicate the increase or decrease of NPY + neurons number at certain depth in injured males and females compared to controls (upper row of each depth section), or in injured females compared to injured males (lower row of each depth section)

**Table 6:**
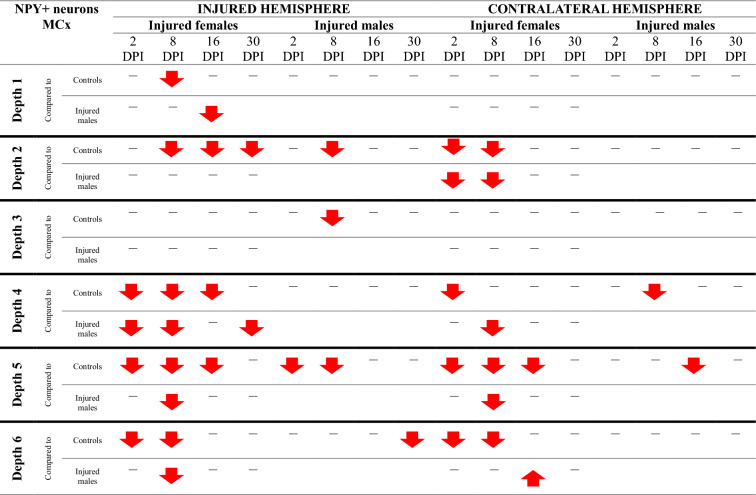
Changes of NPY + neurons density after TBI in motor cortex

The arrows indicate the increase or decrease of NPY + neurons number at certain depth in injured males and females compared to controls (upper row of each depth section), or in injured females compared to injured males (lower row of each depth section)

### nNOS + neurons density increases in somatosensory and motor cortices

Injured females exhibited a significantly higher number of nNOS + neurons in the perilesional somatosensory and motor cortex compared to controls at multiple time points: 2DPI (MCx level 5, p < 0.009), 8DPI (SCx level 4, p < 0.04; MCx level 5, p < 0.02), 16DPI (SCx levels 1, 5, 6: p < 0.04, p < 0.04, p < 0.02, respectively; MCx level 4, p < 0.05), and 30DPI (SCx levels 5, 6: p < 0.02, p < 0.05) (Fig. 15A–D, F–G). In injured males, a similar increase in the number of nNOS + neurons was noted at 16DPI (MCx level 6, p < 0.04) and 30DPI (SCx level 4, p < 0.02; MCx level 5, p < 0.02) relative to controls (Fig. 15G-H). A contralateral increase in nNOS + neuron numbers was observed in injured females compared to controls at 2DPI (MCx level 4, p < 0.04), 8DPI (MCx level 6, p < 0.02), and 30DPI (SCx level 4, p < 0.006) (Fig. 15B, F, H). Injured males demonstrated a similar increase in nNOS + neurons in contralateral cortex compared to controls at 16DPI (MCx level 2, p < 0.05) (Fig. 15E). At 8DPI and 16DPI, injured females exhibited a significantly higher density of nNOS + neurons than injured males (8DPI: MCx level 2, p < 0.03; 16DPI: SCx level 1, p < 0.04) (Fig. 15A, E). The changes in the density of nNOS + neurons after TBI across all cortical layers are presented in Tables 7 and 8.

**Table 7:**
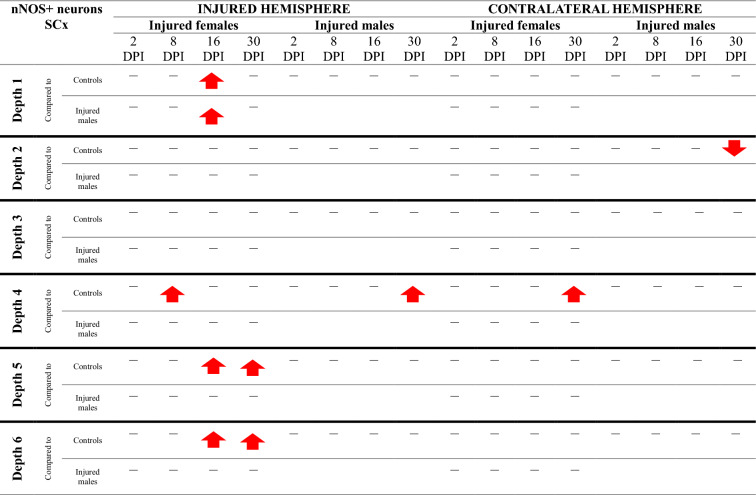
Changes of nNOS + neurons density after TBI in somatosensory cortex

The arrows indicate the increase or decrease of nNOS + neurons number at certain depth in injured males and females compared to controls (upper row of each depth section), or in injured females compared to injured males (lower row of each depth section)

**Table 8:**
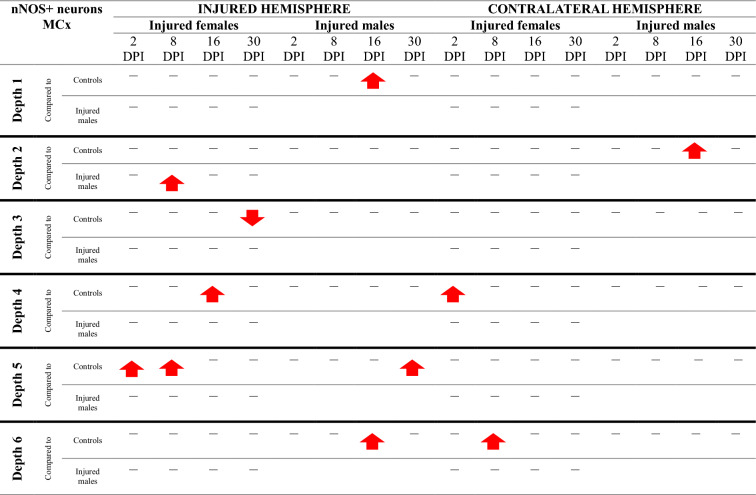
Changes of nNOS + neurons density after TBI in motor cortex

The arrows indicate the increase or decrease of nNOS + neurons number at certain depth in injured males and females compared to controls (upper row of each depth section), or in injured females compared to injured males (lower row of each depth section)

## Discussion

The disparity in brain injury symptoms, outcomes, and development of post-injury disorders between males and females indicates a significant impact of sex on the course of response to TBI. However, mostly male animals have been used so far in the studies conducted on animal models of TBI. The present study is the first to describe the progression of changes occurring in the cerebral cortex following focal injury in both sexes, including the development of a glial scar, as well as changes in the density of various neuronal populations. Sex differences have been observed, both in the glial response to injury and in the degree of neuronal loss.

The analysis of glial scar development revealed strong astrogliosis at 2 and 8 days post-injury, manifested by an increase in the level of the cytoskeletal protein GFAP and hypertrophic morphology of astrocytes surrounding the injury core. GFAP immunoreactivity in the proximity of the lesion decreased gradually by 30DPI, however, it remained higher than in the control groups, suggesting permanent changes in the structure and function of astrocytes forming the glial scar. These findings are consistent with our previous observations in males one month post-injury (Setkowicz et al. 2016) and with the results of Cieri et al. (2023), describing signs of astrogliosis 28 days following stab-wound injury. In accordance with previous reports, by 1 week post-TBI, disrupted BBB is restored by astrocytic processes, and the fibrotic part of the scar is formed by perivascular fibroblasts attracted to the lesion site. The scar starts to maturate during the second week by forming tight borders between fibrotic and glial components, referred to as glia limitans (Kawano et al. 2012). Astrocytes around the injury site undergo morphological changes over time. To date, morphological changes in astrocytes following stab-wound brain injury have been described in males, such as increased cell complexity and processes length, reaching their highest values 7 days post-injury (Cieri et al. 2023). At the same time, increases in cell perimeter, number and length of processes, accompanied by a decrease in cell circularity were observed by Delgado-García et al. (2024). In the case of diffuse TBI in young males (closed-head injury), hypertrophic astrocyte morphology was observed in the cortex for 30 days post-injury, with the most complex morphology, also occurring at 7DPI (Clément et al. 2020). Our observations support the abovementioned findings, as male astrocytes in the perilesional motor cortex at 8DPI exhibited the longest processes, the largest *Convex hull area*, and *Cell area*. Moreover, a contralateral reaction was observed in males at 8DPI, indicating a peak of astrogliosis at this time point post-injury. On the other hand, the increasing cell complexity over the 30-day period in the distal ROI of the motor cortex shows that cells located further from the injury site respond to tissue damage with a certain delay. The most prominent sex differences in astrocyte morphology were observed 2 and 8 days post-injury, when female astrocytes displayed a greater morphological complexity, as indicated by higher values of parameters such as *Cell area*, *Cell density*, *Sum of intersections*, and *Ramification index*. Additionally, the contralateral reaction in females occurred earlier than in males, i.e. 2 days post-injury. This suggests a stronger astrocytic response to injury in females. Furthermore, the GFAP + area fraction in the proximity of the injury site at 30DPI was higher in females compared to males, which may indicate a sustained high GFAP level for a month post-injury. Previous studies investigating astrogliosis levels in both sexes have yielded conflicting results. In the controlled cortical impact (CCI) model, GFAP + area fraction in the cortex was lower in females than in males 1 and 7 days post-injury (Villapol et al. 2017) or higher in females than in males 1 day post-injury (Jullienne et al. 2018), whereas in the stab-wound injury model there was no significant sex difference 7 days after injury (Acaz-Fonseca et al. 2015). In vitro, astrocytes derived from females were more resistant to oxidative stress (Liu et al. 2007), produced less IL-6 (interleukin 6), TNFα (tumour necrosis factor α), and IL-1β (interleukin 1β), but more IP-10 (interferon gamma-inducible protein 10) after LPS (lipopolysaccharide) stimulation (Santos-Galindo et al. 2011), and decrease phagocytic activity in response to inflammation, unlike male-derived astrocytes (Crespo-Castrillo et al. 2020). The aforementioned studies provide evidence of sex differences both in the astrocytic response to homeostasis disruption and in their intrinsic ability to return to physiological functions. These differences may be a result of both the gonad-independent action of sex chromosomes and the interactions with sex hormones, whose receptors show altered expression following TBI (Chowen and Garcia-Segura 2021). The differences that we have observed in the astrocyte morphology and GFAP + area fraction suggest a distinct reactivity of these cells in females and males. Excessive astrogliosis is thought to compromise certain physiological functions of astrocytes (Sofroniew 2009; Zhou et al. 2020). Therefore, we hypothesize that increased astrogliosis in females may negatively impact the neuronal regeneration in the perilesional area. However, determining the consequences of increased reactivity of female astrocytes would require further functional analyses of the cells and the investigations of their secretory activities.

In the present study, an increase in Iba1 immunoreactivity of microglial cells persisting for 30 days was observed. Additionally, a contralateral reaction was noted at 30DPI, indicating a global microglial reaction to the injury. These observations are consistent with the findings of recently published studies using the stab-wound injury model (Cieri et al. 2023). The microglial response was also accompanied by changes in cellular morphology. Classically, there are four morphological categories of microglia: the ramified form with a small, round cell body, considered resting; intermediate forms: hypertrophic, with an elongated cell body, thicker primary processes, and retracting distal branches, and ‘bushy,’ with a large cell body and numerous thick processes; as well as the amoeboid form, without or with few relatively short, thick extensions (Sołtys et al. 2001; Karperien et al. 2013). Recent studies have shown that microglial activation is a continuous process during which cells adopt numerous intermediate forms with varied morphology and function (Vidal-Itriago et al. 2022). Our analysis of microglial morphology revealed an initial activation of cells, demonstrated by a simplified morphology at 2DPI which was followed by a gradual recovery of branched forms, accompanied by increases in the number of their processes and *Cell perimeter*, and decreases in *Cell density* and *Cell circularity.* Sexual differences were also observed. At 2DPI, microglial cells in females exhibited higher *Cell density* and *Cell area*, suggesting a stronger activation compared to males. On the other hand, the higher *Fractal dimension* indicates greater cell complexity, as does the tendency to have a larger perimeter and more processes. Villapol et al. (2017) observed numerous ‘bushy’ and amoeboid cells in males at 1 and 3 days after CCI, which were significantly fewer in females, while the latter exhibited ramified cells with thick processes. This suggests different patterns of morphological changes in male and female cells, with a possible delayed response in females. By analyses of further time points, we have revealed greater *Cell density* and *Cell circularity* in female microglia at 16DPI, along with a tendency for fewer processes compared to males, which may indicate a slower recovery of the branched cell phenotype in females. In contrast, microglial cells in females at 30DPI displayed larger *Cell area*, *Cell perimeter*, and higher *Ramification index*, suggesting a more complex phenotype in females. More ramified microglia may be a typical feature of females. It has been shown that females have more ramified cells in the parietal cortex compared to males on postnatal day 60 (Schwarz et al. 2012). Studies on sex differences in microglial activity following TBI yield conflicting results again. Doran et al. (2019) reported a lower reactive oxygen species production and reduced phagocytic activity of microglia in females after CCI. Barreto et al. (2014) described a decrease in microglial reactivity in ovariectomized females after penetrating brain injury following administration of selective oestrogen receptor modulators, suggesting a protective role of oestrogens. Nevertheless, in another study by Acaz-Fonseca et al. (2015), male rats showed a higher number of non-reactive microglial cells and increased expression of the protective neuroglobin in microglia after stab-wound injury.

In addition to the glial cell response to injury, changes were also observed in specific populations of neurons in the perilesional cortex. Both females and males exhibited a loss of cells expressing parvalbumin and NPY, while the number of nNOS + neurons increased.

So far, a decrease in the number of PV + neurons was observed 2 days post-injury in a controlled cortical impact model (Quiñones et al. 2024), which is in line with our observations in the motor cortex. Additionally, PV + neurons degeneration has also been reported between 2–5 weeks after CCI (Nichols et al. 2018; Cantu et al. 2015; Koenig et al. 2019), which we confirmed by showing a progressive decline in the number of PV + neurons in the somatosensory cortex over 30 days following TBI. A contralateral loss of PV + neurons was observed 3 and 6 weeks post-injury in the fluid percussion injury model (Hameed et al. 2023), which is consistent with our data on contralateral loss of these neurons in females 4 weeks after TBI. The temporal decrease in PV immunoreactivity in the contralateral cortex of males may be explained by a transient decrease in parvalbumin expression without cell death, as noted in males 14–19 days post-CCI (Nichols et al. 2018). In our experiment, the number of PV + neurons one month after injury was significantly lower in females than in males, indicating stronger neurodegeneration in females. Studies on sex differences in the response of PV + neurons to injury are limited. Tucker et al. (2019) observed a loss of PV + neurons in the hippocampus in males after repetitive concussive brain injury, which was not observed in females. Koh (2014) demonstrated neuroprotective effects of oestradiol, which prevented the loss of PV + neurons and the decrease in parvalbumin expression 24 h after ischemic brain injury in ovariectomized females.

In the case of NPY + neurons, we have observed a decrease in their number in the entire cortical thickness within the examined areas, both immediately and 30 days after TBI, with a more prominent loss in females. Previous studies have reported a degeneration of NPY + neurons in the frontal cortex 28 days after fluid percussion injury (Abdul-Muneer et al. 2022), as well as a decrease in NPY expression in the hippocampus 48 h after injury in this model (Bhowmick et al. 2021). In the model of diffuse brain injury, the number of NPY + neurons decreased in the supragranular and infragranular layers of the cortex 24 h after injury, but returned to control values 14 days post-TBI (Carron et al. 2018). The loss of NPY + neurons has also been observed in the hippocampus of rats one month post-CCI, and progressed after the induction of post-traumatic epilepsy (Sun et al. 2018). NPY plays a role in regulating neuronal activity and may have anticonvulsant effects. However, it is also involved in stress and anxiety regulation, with low levels of NPY being noted in anxiety and depressive disorders (Morales-Medina et al. 2010; Ozsoy et al. 2016). It seems that NPY may play a role in the development of various post-traumatic disorders.

Importantly, both for PV + and NPY + neurons, it was previously shown that their loss following TBI was not necessarily related to cell death, but may result from the changes in expression of PV and NPY (Nichols et al. 2018; Carron et al. 2018). The present study does not show the changes in the general neuronal density (e.g. via NeuN staining) or the number of dying neurons (e.g. via Fluoro-Jade C staining), which is a significant limitation. Performing these types of analyses would provide a general idea of neuronal loss in the cortex. Nevertheless, the mentioned tools would not allow identifying the persisting neurons losing PV or NPY immunoreactivity. The explanation of the nature of the loss of PV and NPY immunoreactivity requires the use of genetic tools, such as those used by Nichols et al. (2018) allowing tracking of the cells that expressed PV or NPY at some developmental points and observing the loss and gain of immunoreactivity in these neurons in subsequent time points after TBI. Therefore, although showing an undeniable impact of the brain injury on PV + and NPY + neurons populations in our experiment, we believe that determining the cause of the loss of immunoreactivity requires further investigation.

Nitric oxide is a gasotransmitter involved in the neurotoxicity related to NMDA receptor activation, apoptotic cell death, free radical production, and the exacerbation of inflammation (Wada et al. 1998; Kozlov et al. 2017). Inhibition of nNOS activity or its interaction with the postsynaptic density protein 95 (PSD95) has shown positive outcomes following TBI, such as reduced brain oedema, blood–brain barrier permeability, and reduced apoptosis and lesion size (Sharma et al. 2006). An increase in the number of nNOS + neurons was noted in both ipsilateral and contralateral cortices 5 h after scalpel-incision injury (Sharma et al. 2006). Increases in nNOS expression were observed 2–12 h (Raghavendra Rao et al. 1999), as well as 7 days after CCI (Hall et al. 2012). This prolonged activation suggests that nNOS plays a role in secondary injury development and in the time-dependent changes that occur in the post-traumatic brain. Our data support this hypothesis, as the increases in the number of nNOS + neurons were observed two days after injury, as well as in further time points. Additionally, we observed a higher number of nNOS + neurons in females than in males around the site of injury, as well as an earlier increase in their number. So far, no sex differences have been observed in the basal expression of nNOS (Keser et al. 2011; Singh et al. 2000). However, both male and female sex hormones have been shown to influence nNOS activity (Panzica et al. 2006). Furthermore, in the cerebral cortex of male pigs, 3 h after haemorrhagic shock, there were more nNOS + neurons than in females (Semenas et al. 2010). On the other hand, females exhibited greater hippocampal nNOS activity in response to stress (Keser et al. 2011).

The age of the animals at the time of injury represents a critical variable, primarily due to the influence of sex hormones on tissue responses to traumatic brain injury. Bell (2018) reported that in female rats, oestrogen and progesterone levels begin to rise around P39 and P35, respectively, while testosterone levels in males increase between P40 and P60. The majority of TBI studies utilise adult animals with fully developed endocrine profiles. While sex differences are frequently observed, the results remain inconsistent. Even within studies using the same model—controlled cortical impact (CCI)—findings vary: for instance, Jullienne et al. (2018) reported increased gliosis in females with no sex-related differences in neuronal loss, whereas Villapol et al. (2017) found reduced gliosis and apoptosis in females. These discrepancies suggest that neither male nor female sex hormones exert a consistent or directly neuroprotective effect. Notably, sex-related differences have also been documented in juvenile animals subjected to TBI prior to full sexual maturation, such as on P30 or earlier. Juvenile females exhibited disrupted social behaviour and depressive-like symptoms, whereas males tended to show short-term memory deficits and hyperactivity (Wright et al. 2017; Mychasiuk et al. 2014, 2015). Additionally, greater atrophy in the prefrontal cortex has been observed in females, while males displayed increased microglial proliferation within the hippocampus (Wright et al. 2017; Neale et al. 2023). In the present study, the injury was induced on postnatal day 30, during adolescence. This time point was selected based on prior findings from our laboratory, which indicated significant epileptogenesis and pronounced astrogliosis following this type of injury (Janeczko 1989; Setkowicz and Janeczko 2003). The use of adolescent rats limits the ability to directly assess the effects of sex hormones on early post-injury tissue responses. On the other hand, the study provides evidence that sex differences in injury response can manifest independently of circulating sex hormones and may instead be attributed to genetic sex. The variations observed at later time points, such as P46 and P60, are likely shaped by both chromosomal and hormonal influences, as hormone levels increase during this developmental window. Definitive conclusions regarding the relative contributions of these factors would require studies using hormone-depleted models, such as ovariectomised females or orchidectomised males.

## Conclusions

In the present study, we have demonstrated the occurrence of the structural reorganisation of brain tissue after penetrating injury, including the alterations in the density of specific neuronal populations, and the formation of glial scar, accompanied by the dynamic morphological changes in astrocytes and microglia. Post-injury changes were sex-dependent, including a stronger astrocytic response in females, manifested by an earlier onset of the contralateral reaction and a more hypertrophied astrocytic phenotype around the injury site immediately after TBI. Additionally, the microglial morphology following TBI differed between sexes, including more ramified microglia cells in female rats. Females exhibited stronger activation of nNOS + neurons and more prominent loss of PV + and NPY + neurons. Further experiments are necessary to determine the relationship between glial cells reaction to TBI and the degeneration of specific neuronal populations in each sex, and to understand the mechanisms underlying sex differences in the development of post-traumatic disorders. Moreover, our findings suggest that the efficacy of novel therapies should be tested in both males and females in order to establish the proper therapeutic solutions for each sex.

## Data Availability

Research data are not shared, as they will be employed for comparisons in an upcoming publication that is currently being prepared.
